# A Low-Cost Multimodal Testbed for Array-Based Electrophysiological Microelectrodes

**DOI:** 10.3390/s25092874

**Published:** 2025-05-02

**Authors:** Cat-Vu H. Bui, Neethu Maliakal, Hasan Ulusan, Andreas Hierlemann, Fernando Cardes

**Affiliations:** 1 Bio Engineering Laboratory, Department of Biosystems Science and Engineering, ETH Zurich, 4056 Basel, Switzerlandhulusan@metu.edu.tr (H.U.); andreas.hierlemann@bsse.ethz.ch (A.H.); fernando.cardes@bsse.ethz.ch (F.C.); 2 Department of Electrical and Electronics Engineering, Middle East Technical University, 06800 Ankara, Türkiye

**Keywords:** microelectrode arrays, instrumentation, microfabrication, electrochemical impedance spectroscopy, cellular electrophysiology

## Abstract

Electrode designs and materials have become an increasingly important performance driver for microelectrode arrays, which are among the essential tools for cellular electrophysiology. Ongoing works have continuously innovated over a diverse range of electrode shapes, sizes, and materials. The large design and fabrication parameter space represents rich opportunities for optimizing performance and functionalities as well as a challenge for electrode developers due to a lack of predictive simulation software to aid design works. Electrode prototypes often need to be fabricated, empirically evaluated, and iteratively optimized at significant cost. Efficient hardware testing solutions to aid the development of new electrodes, especially at an early stage when the number of candidate designs is still high, are therefore increasingly important. Here, we propose and implement a cost-effective testbed platform, which is aimed at obtaining first-order characteristics from electrode prototypes to inform early-stage screening and refinement. Upon testing with microfabricated electrodes, the platform was shown to achieve an impedance measurement accuracy comparable to commercial equipment and effectively recorded extracellular action potentials of in vitro rat cortical neurons. By providing relevant electrode testing at a significantly lower cost, in a more compact form, and with greater ease of assembly, compared to existing hardware solutions, the presented testbed can meaningfully lower entry barriers for the development of new array-based electrophysiological microelectrodes.

## 1. Introduction

Microelectrode arrays (MEAs) constitute an important class of tools for cellular electrophysiology, where the electrical properties of electrogenic cells, e.g., neurons or cardiomyocytes, are of central interest. MEAs typically employ multiple closely spaced microscale electrodes capable of recording electrical signals from biological cells. Due to their increasingly diverse and promising applications including basic neuroscience and cardiac research, brain-computer interfaces, drug screening, and precision medicine, efforts in the past few decades have continuously advanced the technology in terms of the number of electrodes, number of parallel recordings, spatio-temporal resolution, signal-to-noise ratio, co-integration of a multitude of sensing and stimulating modalities, ease of use, and throughput [1,2,3]. These advancements have been realized via refinements of all components constituting MEA technology including array design, integrated circuitry, off-chip hardware, and software. While all these components contribute to the final performance of a MEA system, the design of the microelectrodes that make up the array remains crucial. Consequently, electrode engineering, mostly via micro-nano-fabrication techniques, has become an increasingly important path to innovate these systems.

Microelectrode designs and fabrication processes spanning a large number of different materials [4,5,6,7,8], shapes [9,10,11,12,13], and sizes [14,15,16] have been reported and extensively reviewed [17,18,19,20]. The large number of design choices, while allowing for diverse possibilities of novel and performant functional structures, nonetheless, comes at the expense of engineers having to optimize over an increasingly expansive parameter space. This issue is further complicated by a lack of adequate simulation tools to aid design. Consequently, during early-stage development when the number of possible design candidates remains high, electrode engineers have to iteratively fabricate and test multiple designs to empirically screen and optimize.

The necessity of empirical testing during the early-stage design of electrodes incurs significant cost and represents a non-trivial barrier to new electrode development. Therefore, cost-effective hardware solutions aiding new electrode design and development also become increasingly important. In this paper, the preliminary results of which were presented at the Eurosensors conference [21], we first identify what constitutes a useful set of tests for the early-stage screening of array-based electrophysiological recording electrodes and describe the current challenges in implementing these tests. We then address the needs and challenges by implementing a cost-effective, compact, and easy-to-assemble testbed hardware solution that seeks to facilitate a lower entry barrier for and more efficient development of new array-based electrophysiological microelectrodes. Finally, we experimentally demonstrate the testbed with different electrodes microfabricated on 60-electrode passive arrays. At significantly lower costs than existing solutions, the platform can perform electrochemical impedance spectroscopy (EIS) with an accuracy comparable to that of commercial equipment, enabling the resolution of impedance differences between microscale electrodes. Additionally, the platform is demonstrated to record from in vitro cell cultures at signal-to-noise ratios comparable to those of high-cost MEA recording systems, which facilitated the extraction of spike amplitudes from neurons as a test metric.

## 2. Testbed Architecture

### 2.1. Testing Requirements

For electrodes recording from electrogenic cells, signal-to-noise ratio is one of the primary performance considerations, since it dictates what type of biological signals are resolvable and how much information is available to computational routines, such as spike feature extraction, spike sorting, electrical footprint, and cell morphology reconstruction. Electrode impedance inversely correlates with the signal-to-noise ratio and is, therefore, an important predictor of performance [3]. Specifically, EIS is considered a more robust method compared to single-frequency impedance measurements, since biological signals can cover a broad spectral range [22]. Therefore, EIS constitutes a useful first test to evaluate the electrodes in isolation. Secondarily, since the overall performance of a recording electrode is not only a function of its electrical properties but also of how biological cells respond to its microstructures and materials, one needs to evaluate cell–electrode coupling performance as well as biocompatibility. Therefore, a second test is necessary where some electrophysiological signals of interest are recorded from electrodes under test. Specifically, this electrophysiological recording (ER) test aims to measure and report on the amplitudes of action potentials, or spikes, of cells coupled to electrodes. We propose that these two tests provide a useful performance evaluation for the purpose of the early-stage screening of new electrode designs.

Currently, implementing these two tests may incur costs for electrode developers. For impedance testing via EIS, electrode developers usually have to rely on various commercial general-purpose instruments, such as potentiostats or lock-in amplifiers. These instruments tend to be bulky and costly. Additionally, since they are not made for interfacing with MEA devices specifically, they cannot automatically address all electrodes in an array without additional instrumentation from users, reducing testing throughput. For the ER test, the new electrodes often need to be operated by a MEA recording system. Such systems generally host a certain number of costly recording amplifiers. This requirement is necessary for end users, namely electrophysiologists, biologists, and neuroscientists, who need simultaneous recordings across multiple electrodes to investigate multicellular biological systems. This level of functionality is, however, not strictly necessary for electrode engineers, whose primary interest in early-stage electrode development is the performance of individual electrodes featuring different designs. Therefore, these systems are unnecessarily costly for the purpose, with commercial platforms reaching the range of several tens of thousands of euros and cost-optimized do-it-yourself variants costing several thousands of euros [23,24]. A testbed platform that delivers all relevant electrode tests at low cost, in compact form while featuring comparative ease of assembly would allow microfabrication-focused or otherwise budget-constrained groups to extend their areas of interest into electrophysiological electrode works with no great additional investment in infrastructures and expertise.

### 2.2. System Design

**Figure 1:**
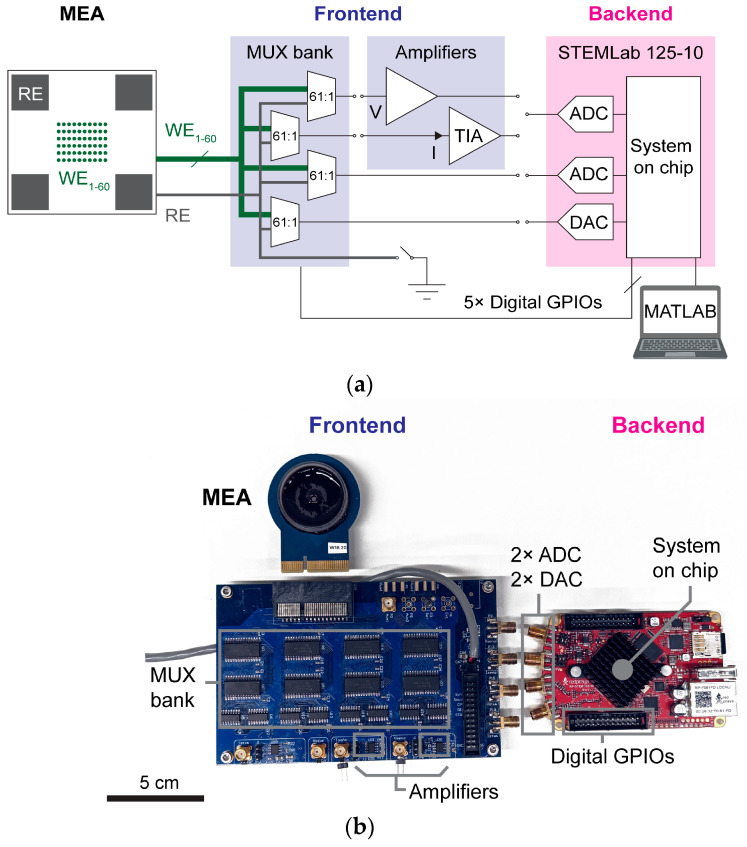
The test platform is depicted as follows: (**a**) a component schematic and (**b**) a physical implementation. The platform consists of a backend board and a frontend board capable of accommodating a passive microelectrode array (MEA) of up to 60 working electrodes (WEs) and 1 reference electrode (RE). The frontend board features a multiplexer (MUX) bank, the outputs of which can be connected to either a low-noise voltage amplifier for voltage readout or a transimpedance amplifier (TIA) for current readout. The STEMLab 125-10 serves as a backend board, providing a system on a chip, up to two digital-to-analog converters (DACs), up to two analog-to-digital converters (ADCs), and general-purpose digital inputs/outputs (GPIOs). The system is controlled via custom MATLAB scripts and the vendor-provided application programming interface library.

To address the requirements outlined previously, we proposed and implemented a new testbed. Partially leveraging the rise of low-cost off-the-shelf single-board microcontrollers/system on a chip, we built our system around the STEMLab 125-10 (Red Pitaya, Solkan, Slovenia). When complemented with a custom frontend board with a relatively small number of electrical components, the platform can perform multiplexing, amplification, signal recording, and generation functions in a versatile and compact form (Figure 1). However, unlike in full-scale MEA systems, we employed a minimum amount of amplifiers and opted for multiplexing up to 60 electrodes, thereby reducing costs while still enabling testing a sufficient number of electrodes at good throughput. The basic functions can be combined into various configurations to deliver two relevant tests for microelectrodes, namely EIS and ER. The test platform can be assembled for costs of approximately 500 euros, a ten- to hundred-fold reduction in costs to alternatives mentioned previously. Moreover, since a large part of the needed electrical functions and codebase are readily provided by the off-the-shelf STEMLab, the testbed also allows for greater ease of assembly.

The frontend board provides multiplexing and amplification functionalities (Figure 1a). This board features a multiplexer (MUX) bank capable of multiplexing up to 61 inputs to 4 output channels. The frontend board can receive a MEA device hosting electrodes under test via a PCIe socket, which connects to the input of the MUX bank. Signals of channels selected by the MUX bank can be amplified by either a voltage amplifier for voltage readout or a transimpedance amplifier (TIA), which acts as a current-to-voltage converter for current readout (Supplementary Figure S1). Output signals of the MUX bank, with or without amplification, can be accessed through a set of four SubMiniature version A (SMA) connectors.

The STEMLab board provides a Zynq 7010 system on a chip (AMD, Santa Clara, CA, USA), up to two digital-to-analog converters (DACs), up to two analog-to-digital converters (ADCs), and general-purpose inputs/outputs (GPIOs) (Figure 1a). These components enable the rest of the functionalities required for the testbed, namely signal recording, stimulus generation, and digital control. The DACs and ADCs are accessible through SMA connectors and can be connected to the outputs of the MUX bank on the frontend through co-axial cables. Digital GPIOs are used to control the switching of the MUX bank on the frontend board. The system on a chip operates the STEMLab and is controlled via custom MATLAB (R2021a) scripts based on the vendor-provided application programing interface (Red Pitaya OS 1.03-701).

Starting from these functional blocks, the testbed could be configured to operate in either EIS or ER mode, allowing for the multimodal testing of electrophysiological microelectrodes of MEA devices.

## 3. Fabrication, Packaging, and Interfacing for Electrodes Under Test

In order to interface the testbed platform frontend to microscale electrodes, we microfabricated passive MEA chips hosting several electrode designs to be tested. These chips were packaged onto an adapter printed circuit board (PCB), which was then connected to the frontend board via a PCIe socket. The packaged devices could be plated with in vitro cell cultures for ER testing.

Depending on the specific electrode designs that developers wish to test, the MEA chip layout and package described in this section may have to be adapted but generally should serve as a versatile test vehicle template for interfacing various electrodes under test to the testbed. The template supports the testing of up to 60 array-based electrophysiological microelectrodes.

**Figure 2:**
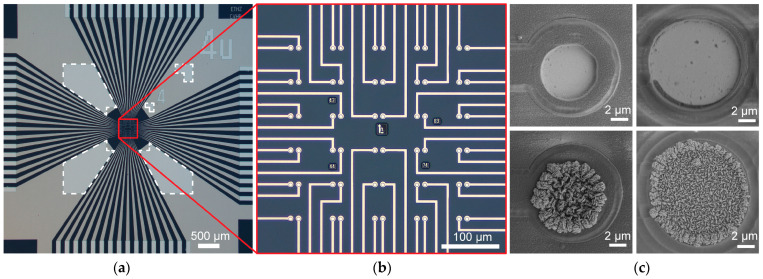
Microfabricated MEAs. (**a**) Optical image of the MEA chip hosting an array of 60 working electrodes (indicated by the red lines) and 1 large reference electrode (indicated by the white dashed lines) on a glass substrate. (**b**) Optical image of the array of 60 working electrodes spanning an area of 310 × 300 µm^2^, consisting of pairs of closely spaced electrodes (14 µm center-to-center distance). (**c**) Scanning electron microscopy images of microelectrodes of nominally 4 µm and 8 µm mask opening diameters (left and right, respectively) and materials of Pt and Pt-black (top and bottom, respectively).

For specific electrodes under test in this paper, we microfabricated circular planar electrodes with photomask openings of 4 µm and 8 µm diameters. Electrodes were made of either platinum or highly porous platinum, known as platinum-black (Pt-black) (Figure 2c). Both high- and low-impedance materials, as well as a small difference in electrode size, were used to effectively show the impedance range and resolution of the platform. Microelectrodes were fabricated on a passive MEA featuring an array of 60 working electrodes (WEs) and 1 large reference electrode (RE) on a glass substrate with SiO_2_ as surface passivation (Figure 2a,b). The sensing array covered an area of 310 × 300 μm^2^ with 30 pairs of electrodes, each with an electrode center-to-center pitch of 14 µm.

Microelectrode arrays were microfabricated using standard cleanroom techniques. Fused silica wafers (100 mm in diameter, 380 µm thick) were used as starting substrates. A layer of 500 nm thick Si_3_N_4_ was first deposited using a Plasmalab 80Plus plasma-enhanced chemical vapor deposition system (Oxford Instruments, Abingdon, UK). Negative photoresist AZ nLOF 2020 (Merck, Darmstadt, Germany) was spin-coated at a spin speed of 5000 rpm. The wafer was then baked at 110 °C for 60 s and exposed with an MA/BA8 Gen4 mask aligner (SUSS MicroTec, Garching, Germany) using an i-line source at a dose of 40–50 mJ/cm^2^. Following a post-exposure bake of 110 °C for 60 s, the wafer was developed for 90 s using an AZ 726 MIF developer (Merck, Darmstadt, Germany). A film stack of Ti-Pt (20/150 nm) was deposited using a BAK UNI evaporator (Evatec, Trubbach, Switzerland). After stripping of the photoresist using a TechniStrip NI555 stripping solution (Technic, Cranston, RI, USA), the metal lift-off process was completed with the Ti-Pt layer patterned to form electrode arrays, tracks, and bond pads. For surface passivation, ~1.2 µm thick SiO_2_ film was deposited using a Plasmalab 80Plus plasma-enhanced chemical vapor deposition system. A second photolithography sequence to define the electrode and bond pad openings was carried out with the same type of photoresist and exposure parameters mentioned in the previous step. Using this photoresist mask, the passivation SiO_2_ was etched through using reactive ion etching (CHF_3_/CF_4_/O_2_) in a Plasmalab 100 system (Oxford Instruments, Abingdon, UK). Photoresist was stripped using O_2_ plasma in a GIGAbatch asher (PVA TePla, Wettenberg, Germany). A blanket SiO_2_ etch was used to ensure over-etching reduced the SiO_2_ film to a final thickness of 1.0 μm. MEA chips were diced from the wafer using a DAD3240 dicer (DISCO, Tokyo, Japan).

For packaging, dies were attached and wire-bonded to adapter PCBs, which provided metal wirings to connect each electrode of the array to the system frontend. The adapter PCB also featured a 3 mm diameter through-hole, which acted as a viewport for observing cells cultured on top of the array using an inverted microscope. After wire-bonding, polycarbonate rings were affixed onto the adapter PCB, and MEA chips were encapsulated by epoxy, leaving only the array exposed, forming a well chamber (Figure 1b).

Pt-black was grown on the electrodes of selected chips by electrodeposition. The chamber was filled with a solution of chloroplatinic acid hexahydrate (7 mM, Merck, Darmstadt, Germany), lead acetate (0.3 mM, Merck, Darmstadt, Germany), and DI water. A platinum wire was immersed in the solution and formed the anode. All the array electrodes were shorted and formed the cathode. A voltage of 1.3 V was applied between the two terminals for ~22 s for microelectrodes fabricated with 4 µm diameter mask openings or ~26 s for microelectrodes fabricated with 8 µm diameter mask openings. Additional deposition time could optionally be added based on visual inspection of Pt-black coatings.

## 4. Electrochemical Impedance Spectroscopy Testing

### 4.1. Electrochemical Impedance Spectroscopy Configuration

**Figure 3:**
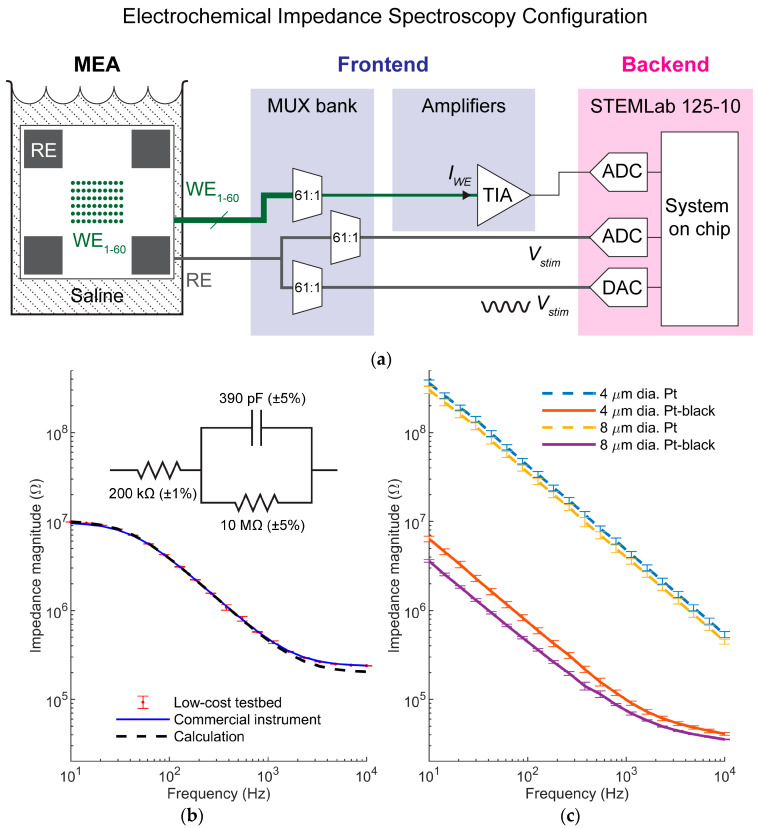
Electrochemical impedance spectroscopy (EIS) test. (**a**) EIS mode configuration. (**b**) EIS measurements obtained by the testbed and a commercial impedance analyzer from the same test load. Mean values of repeated measurements are shown (*n* = 15). Impedance magnitudes of test load were calculated from the values of components (inset). (**c**) Impedance magnitudes of electrodes fabricated with 4 µm and 8 µm mask opening diameters with Pt/Pt-black materials. Mean values of multiple electrodes for each design are shown (*n* = 15). Error bar, where shown, represents one standard deviation in all figures.

For EIS, the system was configured as a two-terminal setup. The on-chip reference electrode was selected via the MUX bank and connected to a DAC on the STEMLab, which acted as the voltage excitation source. This applied voltage excitation signal was simultaneously recorded by connecting the reference electrode via the MUX bank to an ADC on the STEMLab. One working electrode was routed through another output channel of the MUX bank and connected to the TIA. The TIA has a gain feedback resistor of 3 MΩ (Supplementary Figure S1). The feedback resistor was chosen to achieve an optimal balance between low input impedance, sufficient gain, and adequate range, enabling the measurement of hundreds of nA with sub-nA accuracy. The output of this TIA was connected to another ADC on the STEMLab. The STEMlab was configured with a ± 1 V input range. The MEA chamber was filled with a phosphate-buffered saline solution (Thermo Fisher Scientific, Waltham, MA, USA), completing the circuit (Figure 3a). The impedance of this two-terminal system was given by the ratio of the voltage, in complex form, between its terminals to the current, in complex form, flowing through the system. Since the reference electrode was connected in series and was significantly larger than the working electrode, its impedance contribution was considered negligible, and the overall impedance was attributed to only the working electrode under test. Under this assumption, the impedance of the system under test was treated as that of the working electrode.

During a measurement, the DAC generated a series of frequency-varying voltage sine waves with peak-to-peak amplitudes of 50 mV. The excitation signal *V_stim_* was applied to the reference electrode, which was simultaneously recorded by an ADC. The ADC readout was a discrete-time sequence *V_stim_*[*n*]. The TIA measured the current flowing through the working electrode (*I_WE_*), while fixing the working electrode voltage at 0 V. The output of the TIA was digitized by another ADC, from which—after considering the TIA gain—the sequence *I_WE_*[*n*] could be computed. The sampling rate of both ADCs was set to be at least 10 times the excitation frequency in order to avoid aliasing and to allow for sufficient signal resolution for further post-processing. The recording time was set to capture eight periods of the excitation signal. This process could be automatically repeated for each working electrode by programming the MUX bank to loop through all electrodes on the array.

For each measurement, the complex impedance of each electrode under test was extracted in MATLAB using an in-phase and quadrature demodulation calculation. This calculation minimizes the impact of environmental fluctuations and inherent electronic nonidealities, such as thermal noise, offset, and ADC quantization noise. In precise terms, *V_stim_*[*n*] represented the voltage drop across the reference electrode, the electrolyte, and the selected working electrode in series. However, given that the electrolyte resistivity was very low, and that the reference electrode was several orders of magnitude larger than the working electrode, we assumed that *V_stim_*[*n*] was also an accurate estimation of the voltage drop across the working electrode, *V_WE_*[*n*]. In-phase and quadrature (out-of-phase) components, *V*_*WE*,*inphase*_ and *V*_*WE*,*quad*_, were calculated by taking the inner product of *V*_*stim*_[*n*] and reference sinusoids

(1) $$V_{WE,inphase}\approx <V_{\mathrm{stim}}\left[n\right],\cos\left(2\pi f_{o}T_{s}n\right)>,$$

(2) $$V_{WE,quad}\approx <V_{stim}\left[n\right],-\sin\left(2\pi f_{o}T_{s}n\right)>,$$

where *f_o_* was the excitation and reference frequency, *T_s_* was the sampling period, and $<u,v>=\sum_{n=1}^{N}u\left[n\right]v[n]$ denoted the inner product of two vectors of length *N*. Similarly, we calculated the in-phase and quadrature components, *I*_*WE*,*inphase*_ and *I*_*WE*,*quad*_, of the current response as

(3) $$I_{WE,inphase}\approx <I_{\mathrm{WE}}[n],\cos\left(2\pi f_{o}T_{s}n\right)>,$$

(4) $$I_{WE,quad}\approx <I_{WE}[n],-\sin\left(2\pi f_{o}T_{s}n\right)>.$$

The complex impedance *Z_WE_* of the working electrode was then

(5) $$Z_{WE}=\left|Z_{WE}\right|e^{j\varphi t}=\frac{V_{WE,inphase}+jV_{WE,quad}}{I_{WE,inphase}+jI_{WE,quad}},$$

where *|Z_WE_|* and *φ* were the impedance magnitude and phase, respectively.

### 4.2. Impedance Measurements of Electrodes Under Test

To validate the accuracy and repeatability of the testbed, we first compared impedance spectra obtained by the testbed and a commercial impedance analyzer tool (MFIA, Zurich Instruments, Zurich, Switzerland) on the same test load. The test load was constructed by assembling passive electrical components (Figure 3b, inset) into the simplified form of the Randles circuit, commonly used as an equivalent circuit model for the electrode–electrolyte interface [4]. Measurements were performed over a typical electrophysiological range of 10–10^4^ Hz. The test load was connected to the same adapter PCB without any packaging. The commercial tool was connected directly to the outputs of the MUX bank, bypassing the rest of the testbed platform. The MUX bank then routed the test load to the commercial impedance analyzer, also in a two-terminal setup. Measurements were repeated 15 times on the same load for each instrument and impedance magnitude spectra were extracted (Figure 3b). While the testbed exhibited a higher standard deviation compared to the commercial equipment, the means of the impedance magnitudes obtained with each tool closely matched each other. The values obtained with both tools also showed a good match to the calculated values of the test load with a slight deviation at higher frequencies, which most likely originated from the MUX board. While these non-idealities represent limitations of the testbed, the system provided sufficient accuracy and repeatability to evaluate microscale electrodes at the frequencies of interest. The performance was achieved with more than a twenty-fold reduction in costs compared to the specific commercial equipment in this case, demonstrating the cost-effectiveness of the platform as an important advantage.

For the electrodes under test described in the previous section, measurement frequencies were also chosen to be in a range of 10–10^4^ Hz with 20 frequency points at equal distance from each other on the log scale. For each of the electrode designs, the measurements were repeated for 15 electrodes. Impedance magnitude plots (Figure 3c) showed that the electrode impedances slightly decreased as the sizes increased, whereas the Pt-black coating decreased impedance by 1–2 orders of magnitude for the same sizes, in line with previous reports [14]. The impedance reduction stemmed from the high porosity of Pt-black, which significantly increased the surface areas of the electrodes while preserving their footprints. Moreover, while electrode impedance characterization often focuses on reporting impedance only at 1 kHz, which is the representative frequency in the action potential band, impedance spectroscopy measurements across an electrophysiologically relevant range of frequencies, i.e., EIS, convey the frequency-dependent filtering behaviors of the different electrode designs. This information in turn enables designers to evaluate which electrodes are more appropriate for signals of different spectral content (e.g., extracellular action potential (EAP) band includes higher frequencies at 300–5000 Hz while the local field potential (LFP) band includes lower frequencies between 1 and 300 Hz [14]). Specifically, from the impedance magnitude plot (Figure 3c), one can estimate that Pt-black electrodes transitioned from predominantly capacitive to resistive behavior at around 1 kHz, and, therefore, could potentially record EAPs more faithfully compared to Pt electrodes, which exhibited mostly capacitive behavior across the measurement frequency range.

## 5. Electrophysiological Recording Test

While EIS is useful for testing electrodes decoupled from the rest of the recording chain, different biological cell cultures or tissues may exhibit different responses to electrode materials and geometries. Biocompatibility and adhesion affinity between the cells and the electrodes contribute to cell-electrode coupling performance. Therefore, the platform was designed to perform a second test, namely electrophysiological recording (ER), in which the platform was configured to record signals of interest from different cell types and plating protocols. This test was aimed at verifying that electrophysiological signals could be obtained from electrodes under test and yielded signals or detectable spike amplitudes.

It should be noted that the platform delivered this functionality as an efficient test to aid electrode designers to evaluate cell-electrode compatibility and was not intended as a replacement for a full MEA recording system. Specifically, the testbed enabled looping through all electrodes in the array and recording from one electrode at a time. For each electrode, the recording was performed for the entire specified duration before moving to the next electrode in the array. Additionally, due to the buffer size limitations of the off-the-shelf STEMLab, recording for each electrode was performed repeatedly in segments of continuous 1.07 s of measurements. These design choices allowed the system to optimize for cost and ease of assembly while remaining sufficient to provide useful information on electrode performances in the presence of target cell and tissue types. To demonstrate ER functionality, we used Pt-Black electrodes fabricated with 8 µm diameter mask openings to record EAPs from primary rat cortical neurons.

### 5.1. Electrophysiological Recording Configuration

**Figure 4:**
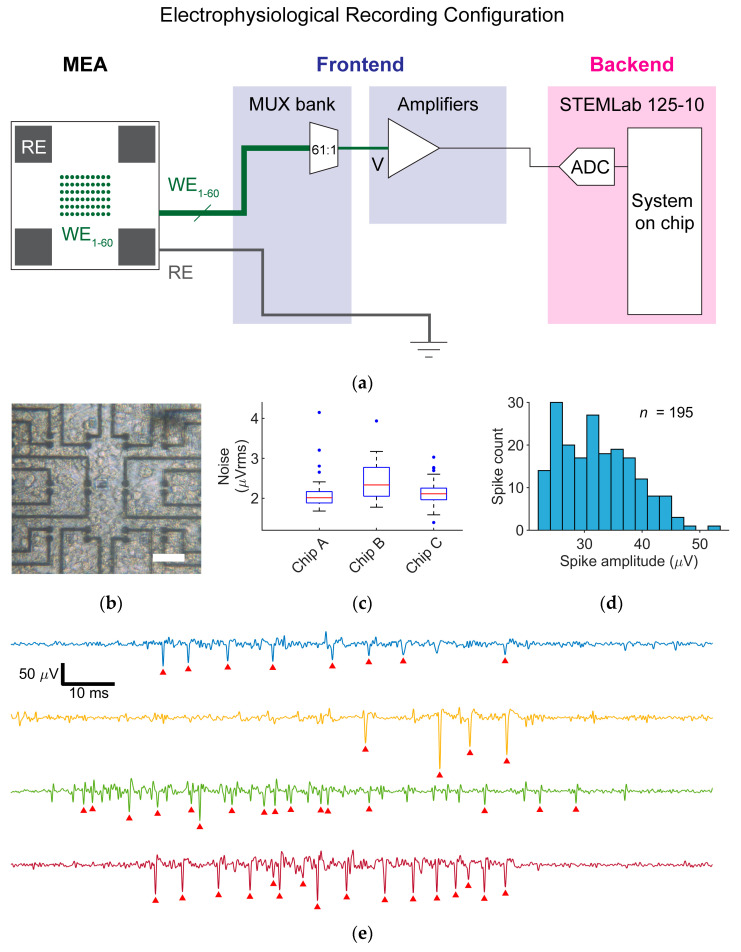
Electrophysiological recording (ER) test. (**a**) ER mode configuration. (**b**) Optical image of a rat cortical neuron culture (DIV 27) on an electrode array. Scale bar is 50 μm. (**c**) Input-referred noise (*V*_*noise*,*rms*_) estimation for Pt-black electrodes with 8 µm mask opening diameters on three chips during electrophysiological recording (*n* = 60 for each chip). (**d**) Histogram of extracellular action potential amplitudes (*n* = 195) on a representative recording of ~10 min from an electrode detecting electrophysiological activity (DIV 31). (**e**) Representative voltage traces (DIV 30–31) recorded from different electrodes on three chips showing extracellular action potentials marked by red triangles.

For the ER mode, one working electrode was routed via the MUX bank to the voltage amplifier (Figure 4a). Output from the amplifier was recorded by an ADC on the STEMLab. The reference electrode was grounded. The maximum continuous recording duration was limited by the data acquisition buffer size, *Buffer* = 16,384 samples, of the STEMLab. For a sampling frequency *f_s_*, the system could record a maximum duration of $Buffer/f_{s}$ before having to save the data and repeating the data acquisition for any total (non-continuous) duration. Using the MUX Bank, the recording could be repeated for any set of electrodes in sequence. By looping through the electrodes one at a time, electrodes detecting electrophysiological activities could be identified. Active electrodes of interest could then be recorded for a longer total duration, from which spike amplitude information could be extracted and reported.

### 5.2. Recording of Neural Extracellular Action Potentials

Rat cortical neurons were plated on MEA devices featuring Pt-black electrodes with an 8 µm mask opening diameter (Figure 2c, bottom right). Before neuron plating, devices were sterilized in 70% ethanol for 40 min and washed three times with sterile deionized (DI) water. The exposed arrays on the devices were then first coated with 7 µL of 0.05% (*v*/*v*) polyethylenimine (Merck, Darmstadt, Germany) in borate buffer (Thermo Fisher Scientific, Waltham, MA, USA) and kept in an incubator for 40 min at 37 °C. After rinsing three times with DI water, the arrays were subsequently coated with 7 μL of 0.02 mg mL^−1^ laminin (Merck, Darmstadt, Germany) in Neurobasal medium (Thermo Fisher Scientific, Waltham, MA, USA) and kept in an incubator for at least 35 min. Cortices of E18 Wistar rat embryos were harvested in ice-cold Hanks’ balanced salt solution (Thermo Fisher Scientific, Waltham, MA, USA) and then dissociated in trypsin with 0.25% ethylenediaminetetraacetate (Thermo Fisher Scientific, Waltham, MA, USA). After cell counting, laminin was removed and 7000 cells were seeded onto the arrays. The devices were then incubated at 37 °C for ~40 min before 2 mL of plating medium was added. The plating medium was taken from a solution of 450 mL Neurobasal medium, 50 mL horse serum (Thermo Fisher Scientific, MA, Waltham, USA), 1.25 mL GlutaMAX (Thermo Fisher Scientific, Waltham, MA, USA), and 10 mL B-27 (Thermo Fisher Scientific, Waltham, MA, USA). Twice a week, 50% of the culture medium was exchanged with a growth medium. The growth medium was taken from a solution of 49 mL BrainPhys medium (STEMCELL Technologies, Vancouver, BC, Canada), 1 mL SM1 (STEMCELL Technologies, Vancouver, BC, Canada), and 100 µL Penicillin–Streptomycin (Thermo Fisher Scientific, Waltham, MA, USA). The cultures were maintained in an incubator at 37 °C and 5% CO_2_. All recordings were performed at 37 °C. A representative image of a neuronal culture on an array taken through the backside viewport of the PCB is shown in Figure 4b.

For all recordings from neuronal cultures, the sampling rate was set to 15.26 kHz, which, as described previously regarding the buffer size of the STEMLab, allowed for a maximum continuous recording duration of 1.07 s. For each chip, all 60 electrodes were recorded for a total (non-continuous) duration of 10.7 s each to scan for electrodes detecting electrophysiological activity. Active electrodes of interest were then selected and recorded for up to 10 min. Input-referred signals were computed by adjusting the ADC output by the gain (× 430) of the voltage amplifier. Signal traces were then bandpass-filtered over 300–5000 Hz. Input-referred noise (*V*_*noise*,*rms*_) was estimated for each electrode by taking the standard deviation of all filtered voltage traces recorded by the electrode. Noise data was obtained in the presence of active cell cultures to include contributions from electrode, instrument-related, and biological noise sources. Signal attenuation across the electrode was assumed to be small for this range of electrode impedance and hence neglected in calculations. EAPs were detected and counted using a spike detection threshold of 10 × *V*_*noise*,*rms*_.

Specifically, we recorded from six MEAs with 8 µm diameter Pt-black electrodes on days in vitro (DIV) 30–31, three of which showed electrophysiological activities. Input-referred noise distributions for three chips with active cell cultures are displayed in Figure 4c and show reasonable electrode-to-electrode and chip-to-chip variability. Among the three chips, 40/180 electrodes were detecting electrical activity. Representative voltage traces containing EAPs, recorded from different electrodes on three chips, are shown in Figure 4e. From a representative longer recording of 10 min from an active electrode, EAPs were extracted with a mean amplitude of 32.3 ± 6.4 µV. A histogram of EAP amplitudes is shown in Figure 4d. The amplitudes of detected EAPs in this case verified the compatibility and adhesion affinity of a typical primary neuronal culture with the specific size and material choice of the electrodes under test. The capability to resolve a large number of EAP signals at signal-to-noise ratios > 10—well within the typical performance of higher-cost full MEA recording systems [3]—demonstrates the cost-effectiveness of the platform for the purpose of capturing spike amplitude statistics as test metrics.

## 6. Conclusions

We designed and demonstrated a low-cost, compact, and easy-to-assemble multimodal testbed for array-based electrophysiological electrodes. At a fraction of the costs of comparable commercial equipment, the system could accurately resolve impedance differences between electrodes of different microscale sizes and material choices across a frequency range relevant to many types of electrophysiological signals. The platform was also used to record EAPs from rat cortical neurons with signal-to-noise ratios comparable to those obtained with higher-cost MEA recording systems. By cost-effectively enabling these two tests, the testbed can aid the early-stage development of electrophysiological microelectrodes and lower the entry barrier to MEA development.

While we have proposed the two aforementioned tests as broadly useful for electrophysiological electrodes, there might be additional testing requirements, which are important for specific MEA functionalities and applications and are not included here. For example, in case the MEA needs to be implantable, the stability of electrodes against degradation over time is a major consideration. While long-term stability testing is not included here, the platform can be readily incorporated into an accelerated aging procedure, where impedance can be tracked over time under conditions of interest as a way to predict reliability during chronic applications. Additionally, the modular design of the platform also addresses the need for testing versatility. The platform can be extended by additional instruments or wiring configurations. For example, a voltage waveform generator can be connected and routed to electrodes to perform electroporation. Lastly, when considered in conjunction with other reports for low-cost methods for making MEAs [25,26,27], our solution may also contribute effectively to bringing cellular electrophysiology and MEA technology to a broader community of not only device and application developers but also educators and students.

## Data Availability

Data is contained within the article or Supplementary Material.

## Supplementary Materials

The following supporting information can be downloaded at: https://www.mdpi.com/article/10.3390/s25092874/s1, Figure S1.
