# Supplementary material for: A Low-Cost Multimodal Testbed for Array-Based Electrophysiological Microelectrodes
Source: Sensors (Basel). 2025 May 2;25(9):2874. doi: 10.3390/s25092874 (PMC12074398; doi:10.3390/s25092874)
Supplement: Supplementary file 1 [file sensors-25-02874-s001.zip › sensors-3564824-supplementary.pdf]

Supplementary Information

A Low-Cost Multimodal Testbed for Array-Based Electrophysiological Microelectrodes

Cat-Vu H. Bui, Neethu Maliakal, Hasan Ulusan, Andreas Hierlemann and Fernando Cardes

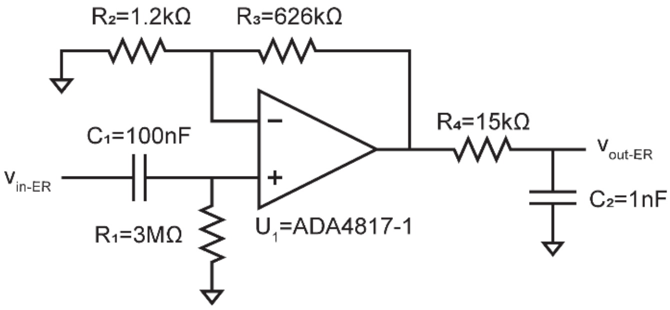

(**a**)

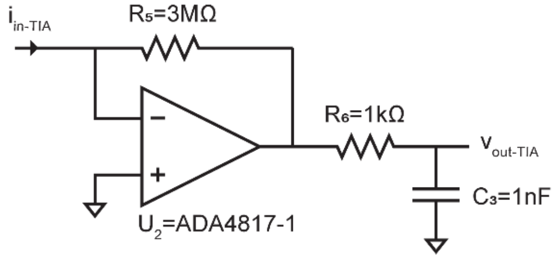

(**b**)

Supplementary Figure S1. (**a**) Simplified schematic of the voltage amplifier in the electrophysiological recording subsystem. A high-pass filter (C_1, R_1) with a cutoff frequency of 0.5 Hz was included to eliminate low-frequency fluctuations. The signal was then amplified by a non-inverting amplifier with an effective gain of 430. This gain was lower than the ideal gain, expected for this topology, due to the limited open-loop gain of the selected operational amplifier. Finally, a low-pass filter (R_4, C_2) with a cutoff frequency of 10.6 kHz was added after the amplification stage to limit aliasing. (**b**) Simplified schematic of the transimpedance amplifier in the electrochemical impedance spectroscopy subsystem. The feedback resistor R_5 defines the gain of the transimpedance amplifier.
